# Relationship of the Circulating Endothelial Progenitor Cells to the Severity of a Coronary Artery Lesion in Unstable Angina

**DOI:** 10.1155/2022/9619626

**Published:** 2022-07-05

**Authors:** Cheng Xiao, Lixiang Liu, Xi Li, Xiaoan Yang, Hanxiong Liu

**Affiliations:** ^1^Medical College, Hunan Polytechnic of Environment and Biology, Hengyang 421005, China; ^2^Department of Gynecology, The Seventh Affiliated Hospital, Sun Yat-Sen University, Shenzhen 518107, China; ^3^Pharmacy Department, Shenzhen Qianhai Shekou Free Trade Zone Hospital, Shenzhen 518067, China; ^4^Department of Infectious Diseases, The Third Affiliated Hospital, Sun Yat-Sen University, Guangzhou 510630, China; ^5^Department of Gastroenterology, The First People's Hospital of Chenzhou, Chenzhou 423001, China

## Abstract

The number and function of circulating endothelial progenitor cells (EPCs) decreased in stable coronary artery disease. Nevertheless, there were few studies that explored the variation of EPC and the relationship with the severity of coronary artery lesions in unstable angina (UA). Therefore, this leaves an area for the investigation of the difference in the number and activity of circulating EPCs and the relationship with the Gensini score in unstable angina. Fluorescence-activated cell sorter analysis, as well as DiI-acLDL and lectin fluorescent staining measure the number of circulating EPCs. The transwell chamber assay and MTT were evaluated by the migration and proliferation of circulating EPCs. In addition, the flow-mediated dilation (FMD), Gensini score, and IL-6 levels in plasma were determined. We found that UA patients had the higher number and lower function of circulating EPCs. With the increase in severity of coronary artery lesions, the migration and proliferation of EPCs were decreased. Moreover, the function of the circulating EPCs was negatively associated with severity of coronary artery lesions in unstable angina. In addition, UA patients presented elevated IL-6, which was negatively correlated with the function of circulating EPCs and FMD and positively correlated with the severity of coronary artery lesions evaluated by the Gensini score. These findings revealed the decline in the function of circulating EPCs was associated with the severity of coronary artery disease, which may be related to systemic inflammation.

## 1. Introduction

Vascular endothelial injury and endothelial dysfunction are the initiating factors of coronary atherosclerosis. Clinical studies have shown that the combined action of risk factors such as hypercholesterolemia and hypertension leads to endothelial injury and repair imbalance, promotes the development of atherosclerosis, leads to coronary artery stenosis and occlusion, and finally leads to heart disease with myocardial ischemia, hypoxia, and necrosis [1–3]. The Gensini score is a very effective method to evaluate the severity of coronary artery disease. Studies have confirmed that the Gensini score has widely been used to explore the correlation between the severity of coronary artery lesions and some clinical indexes [4–6].

Endothelial progenitor cells (EPCs) are pluripotent stem cells derived from the bone marrow. After entering the blood, they eventually differentiate into mature vascular endothelial cells and participate in the process of angiogenesis and repair after endothelial injury [7, 8]. When the vascular endothelium is injured, EPCs are mobilized and released from the bone marrow to peripheral circulation to accelerate vascular re-endothelialization through chemotaxis, adhesion, migration, and proliferation, which plays a key role in the repair of vascular endothelium injury. A large number of studies have confirmed that cardiovascular risk factors and patients with coronary heart disease can lead to different degrees of vascular endothelial injury, and the effect on peripheral circulation EPCs is inconsistent [9]. The more risk factors, the more obvious the decline in the number and function of EPCs, suggesting that the lower function of repairing endothelial injury [10]. In patients with coronary heart disease, the mobilization of peripheral EPC was also inconsistent. In patients with stable angina pectoris, the number and function of EPC decreased, while in patients with unstable angina pectoris, the mobilization of peripheral EPC increased [11–13]. However, there are few studies on the relationship between peripheral circulation EPC function and coronary artery severity in patients with unstable angina pectoris.

Inflammatory response is the key to the occurrence of atherosclerosis. When endothelial progenitor cells are inflamed, in order to reduce the damage caused by them, the body will trigger induced cells to promote the apoptosis of endothelial progenitor cells, leading to the migration of T cells, monocytes, platelets, and the proliferation of smooth muscle cells and then leading to the formation of atherosclerosis [14]. A large number of studies have shown that interleukin-6 (IL-6) plays an important role in regulating the adhesion, migration, and proliferation of endothelial progenitor cells and mediating the repair of vascular injury [14]. Therefore, we hypothesized that the function of EPCs in patients with unstable angina pectoris is related to the degree of coronary artery disease, which may be caused by the increase in IL-6. In order to test this hypothesis, this study detected the function of circulating EPCs and endothelial function evaluated by flow-mediated dilatation (FMD) of unstable angina pectoris, compared the changes of the different Gensini scores, analyzed its correlation with the Gensini score, and discussed the possible mechanism.

## 2. Methods

### 2.1. Characteristics of Subjects and Method

In this study, we enrolled 60 UA patients and 20 controls and their age was >18 years. Inclusion criteria were as follows: UA group: (1) the patients had typical angina attack within 1 month before admission; (2) the ECG scan showed ST-segment descending; (3) the left main artery, left anterior descending artery, left circumflex artery, and right coronary artery were the main blood vessels, and at least two orthogonal projection post urography showed that the diameter of the main blood vessels was narrowed ≥50%. Control group: the normal coronary artery was confirmed by selective coronary angiography. Exclusion criteria were as follows: (1) taking statins and other drugs that may affect circulation of EPC for more than 2 weeks before operation; (2) acute myocardial infarction, tumor, other heart diseases such as chronic cardiac insufficiency, infectious diseases, and liver and kidney dysfunctions; (3) the patient who previously received coronary intervention or coronary artery bypass surgery. The protocol was approved by the ethical committee of our hospital. The basic characteristics of enrolled patients are shown in Table 1.

The degree of the coronary artery lesion and the grouping degree of coronary artery stenosis were quantitatively evaluated by the percentage of reduction of a coronary artery diameter with reference to an angiography catheter or finger guide tube and QCA system software. The Gensini integral method was used to evaluate the degree of coronary artery disease. The quantitative score is as follows: stenosis ≤25% is 1 point, 25%∼50% is 2 points, 51%∼75% is 4 points, 76%∼90% is 8 points, 91%∼99% is 16 points, and 100% is 32 points. For multiple stenoses of a single vessel, the narrowest place was used as the score. Different coronary arteries should also be multiplied by corresponding coefficients, which are left main artery lesions, respectively ×5. The lesion of the left anterior descending branch is the proximal ×2.5 middle section ×1.5 far section ×1. The diagonal branch lesion is *D*1 × 1, *D*2 × 0.5, and the lesion of the left circumflex branch was proximal ×2.5. Both distal and posterior descending branches ×1 posterior branch ×0.5, the lesions of the right coronary artery were proximal, middle, distal, and posterior descending branches ×1. The score of coronary artery stenosis was the sum of the scores of each branch.

According to the Gensini score, the coronary heart disease group was divided into three subgroups: low-risk group: Gensini score ≤30; medium-risk group: Gensini score 31∼59; severe-risk group: Gensini score ≥60.

### 2.2. Cell Culture Test to Evaluate the Number of Circulating EPCs

Blood samples were collected from enrolled patients. The isolation and culture of EPCs are described. The number of circulating EPCs was evaluated by the ratio of CD34+ KDR+ cells per 100 peripheral blood mononuclear cells as previously described. The number of cultured EPCs was also evaluated by DiI-acLDL/lectin double-positive cells/200 and counted manually by two independent observers blinded to the study.

### 2.3. EPC Migration and Proliferation Test

EPC proliferation was determined by 3-(4, 5-dimethylthiazol-2-yl)-2,5-diphenyltetrazolium bromide. After 7 days of culture, EPCs were digested with 0.25% trypsin and then transferred to a serum-free medium in a 96-well plate (200 ul/well). After 24 hours of culture, EPCs were supplemented with 10 *μ*l MTT (5 g/L; Fluka, Sigma-Aldrich, St. Louis, Missouri, USA) and incubated for 4 hours. Then, the supernatant was discarded, and 200 *μ*l dimethyl sulfoxide was added by shaking it for 10 min before measuring the optical density at 490 nm.

EPC migration is carried out by using a modified Boyden chamber. 2 *∗* 10^4^ EPCs were placed in the upper chamber of the modified Boyden chamber. The incubator was placed in a 24-well culture dish containing EBM and human recombinant VEGF (50 ng/ml). After incubation at 370°C for 24 hours, the lower side of the filter was cleaned with PBS and then fixed with 4% paraformaldehyde. Cell nuclei were stained with DAPI for quantification. Then, the cells that migrated to the lower chamber were manually counted by two independent experimenters in three random microscope fields.

### 2.4. Measurement of Plasma Levels of IL-6

Plasma levels of IL-6 were measured by the high sensitivity enzyme-linked immunosorbent assay (*R* & *D* Systems, Wiesbaden, Germany) as previously described [15].

### 2.5. Evaluating FMD

In brief, brachial artery FMD was measured by high-resolution ultrasonography using a 5–12 MHz linear transducer on an HDI 5000 system (Washington, USA) as previously described [16].

### 2.6. Statistical Analysis

The statistical software was SPSS VII.0 (SPSS Corporation, Chicago, Illinois, USA). All the data were presented as the mean SD. Statistical significance was evaluated using Student's *t*-test or analysis of variance. Univariate correlations were calculated using Pearson's coefficient (*r*). *P* < 0.05 was considered statistically significant.

## 3. Result

### 3.1. Baseline Characteristics

Baseline characteristics and laboratory findings of the control and UA groups are listed in Table 1. There was no significant difference in age, BMI, blood pressure, heart rate, AST, ALT, and GLU found in the two groups (*P* > 0.05). The FMD in UA patients was lower than that in the control group (*P* < 0.05). However, the plasma IL-6 level in the UA group was significantly higher than that in the control group (*P* < 0.05).

### 3.2. EPC Number and Function between UA Patients and Control

**Figure 1:**
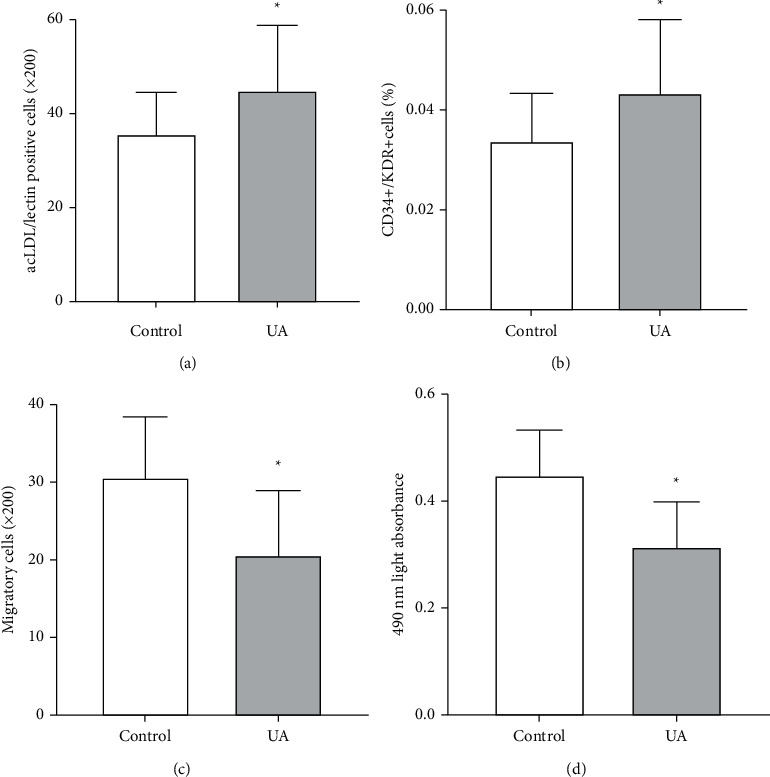
The number and function of circulating EPCs between the two groups. The number of circulating EPCs was detected by determining the number of CD34+/KDR+ cells per 100 peripheral blood mononuclear cells and then by examining the numbers of DiI-acLDL/lectin double-positive cells (a), (b) The results of migration and proliferation assays (c), (d) Statistical significance was evaluated using Student's *t*-test or analysis of variance.^*∗*^*P* < 0.05

The circulating EPC number (a-b) decreased in patients with UA compared with control (Figures 1(a) and 1(b), *P* < 0.05). Compared with the control group, the migration and proliferation of circulating EPCs decreased in UA patients (Figures 1(c) and 1(d), *P* < 0.05).

### 3.3. EPC Number and Function between Three Subgroups

**Figure 2:**
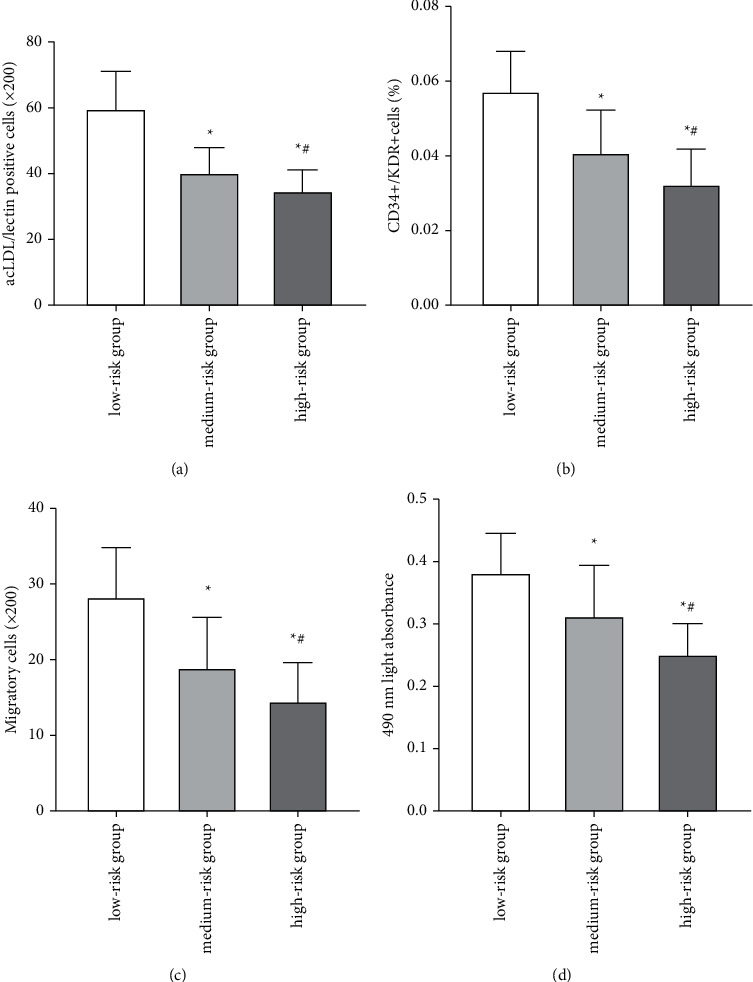
The number and function of circulating EPCs between three subgroups. As the Gensini score increased, the number of circulating EPCs was decreased (a), (b) and the migration and proliferation of circulating EPCs were also declined. (c), (d) Statistical significance was evaluated using Student's *t*-test or analysis of variance.

As the Gensini score increased, the number of circulating of EPCs was decreased (Figures 2(a) and 2(b), *P* < 0.05), and the migration and proliferation of circulating of EPCs were also declined (Figures 2(c) and 2(d), *P* < 0.05).

### 3.4. Correlation between EPC Function and FMD with Severity of Coronary Artery Lesions

**Figure 3:**
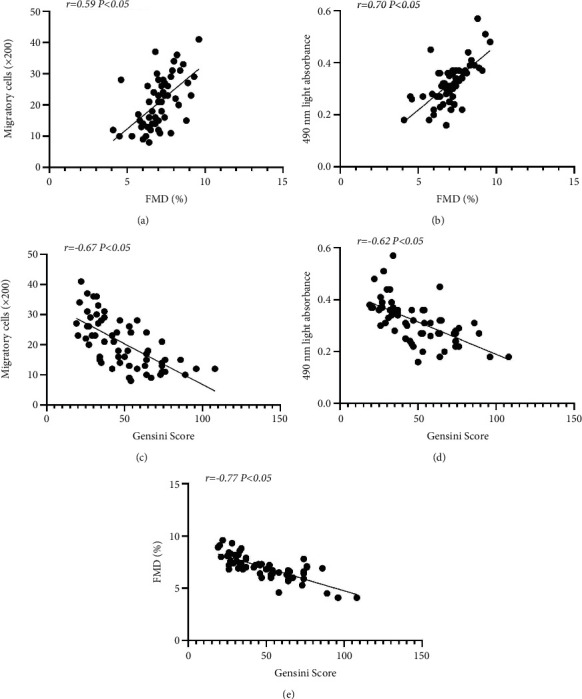
Correlation between FMD and the Gensini score and EPC function. FMD was positively correlated with EPC migration (a) and proliferation (b) In addition, the Genisini score was inversely related to EPC migration (c) and proliferation (d) And the Genisini score was inversely related to FMD (e) Univariate correlations were calculated using Pearson's coefficient (*r*).

As shown in Figures 3(a) and 3(b), FMD was positively correlated with the migration and proliferation of circulating EPCs (Figures 3(a) and 3(b), *P* < 0.05). In contrast, the Gensini score was negatively correlated with FMD and migration and proliferation of circulating EPCs (Figures 3(c)–3(e), *P* < 0.05).

### 3.5. Correlation between EPC Function and FMD with Severity of Coronary Artery Lesions and IL-6

**Figure 4:**
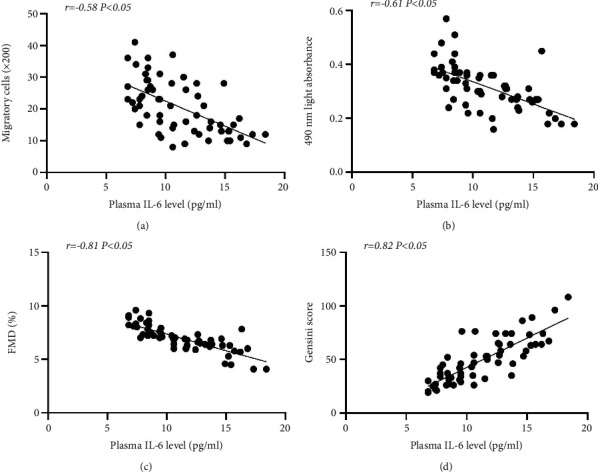
Correlation between the function of circulating EPCs or FMD or the Gensini score and IL-6. EPC migration (a) proliferation (b) and FMD (c) were inversely related to IL-6. In addition, the Gensini score was positively correlated with IL-6 (d) Univariate correlations were calculated using Pearson's coefficient (*r*).

As shown in Figures 4(a) and 4(b), the migration and proliferation of circulating EPCs were negatively correlated with IL-6 (Figures 4(a) and 4(b), *P* < 0.05). Moreover, FMD was also inversely related to IL-6 (Figure 4(c), *P* < 0.05). In contrast, the Gensini score was positively correlated with IL-6 (Figure 4(d), *P* < 0.05).

## 4. Discussion

The present study demonstrated that UA patients had the higher plasma IL-6 level and the number of circulating EPCs. In contrast, UA patients had lower FMD and the migration and proliferation of EPCs than those in the control group. With the increase in the Gensini score, the migration and proliferation of endothelial progenitor cells decreased. In addition, the function of the circulating EPCs was positively associated with FMD. And severity of coronary artery lesions in unstable angina was negatively associated with FMD and the EPC migration and proliferation. Moreover, UA patients presented elevated IL-6, which was negatively correlated with the function of circulating EPCs and FMD and positively correlated with the severity of coronary artery lesions evaluated by the Gensini score. These findings revealed the decline in the function of circulating EPCs was consistent with endothelial dysfunction, and the mitigated endothelial repairability was associated with the severity of coronary artery disease, which may be related to systemic inflammation.

Several lines of evidence obtained in clinical research have demonstrated that the circulating EPC level was declined in the accumulation of cardiovascular risk factors, which was significantly related to endothelial dysfunction [16–18]. However, peripheral circulating EPC mobilization is inconsistent in patients with coronary heart disease. Previous investigations showed that the number of circulating EPCs was decreased in stable CAD but enhanced in acute myocardial infarction [18, 19]. Similarly, the EPC level was significantly higher in unstable angina patients compared with stable angina patients [20], suggesting that necrosis due to infarction is not requisite for their peripheral mobilization. However, there are very few reports concerning the function of circulating EPCs in UA. Our results showed that the migration and proliferation of EPCs in UA patients were lower than those in control. Interestingly, our research revealed that FMD was significantly reduced in UA patients and positively correlated with EPC function; it suggested that although EPC numbers were increased in these patients with UA, vascular endothelial function was decreased.

Circulating EPCs are essential for repairing endothelial injury in coronary artery disease [15]. With the aggravation of coronary artery lesions, the EPC function decreased gradually, and endothelial function injury aggravated gradually, suggesting the severity of coronary artery lesions may be associated with a decline in EPC functions. Increasing evidence suggests that the Gensini score is a common method for estimating the severity of coronary artery lesions of STEMI, NSTEMI, unstable angina, and anginal syndrome [21, 22]. It has a wide range of clinical applications. In the present study, we found that circulating EPC activity was significantly negatively correlated with the Gensini score. The results showed that subdued endogenous endothelial repair capacity accelerated the progression of pathogenesis in UA patients. Therefore, the impairment of endothelial progenitor cells may contribute to evaluating the degree of coronary artery lesions in UA patients.

IL-6 has been shown to be involved in inflammatory processes and in the development and progression of atherosclerosis [23]. Previous studies have demonstrated that the level of IL-6 in patients with ACS is significantly higher than that in patients with stable angina pectoris [23]. Here, we similarly observed the higher plasma IL-6 level in unstable angina relative to those in the control group. In addition, several studies have demonstrated that a correlation exists between IL-6 levels and the severity of coronary artery lesions in CAD [24, 25]. Similarly, in the present study, we demonstrated that positive association between IL-6 levels and the severity of coronary artery lesions were evaluated by the Gensini score in unstable angina. Indeed, we also reported a negative relation between IL-6 levels and the function of circulating EPCs and FMD. These results suggested that IL-6 may be the potential mechanism underlying hypofunction of circulating EPCs, which may be a potential biomarker for evaluation of coronary artery lesions of unstable angina.

The findings presented in this study have important clinical implications. Firstly, the impairment of circulating EPC function and endothelial dysfunction in patients with unstable angina pectoris are negatively correlated with the Gensini score, indicating that circulating EPC and FMD may be an important biomarker for evaluation of severity of coronary artery lesions in unstable angina. Secondly, IL-6 may be partly responsible for the hypofunction of circulating EPCs and severity of unstable angina patients. Anti-inflammation treatment may be beneficial to unstable angina patients [26].

Some limitations should be acknowledged. The enrolled patients were not enough. Furthermore, a large-sample study needs to be carried out to explore the potential predictive value of circulating EPCs in UA. Moreover, our study is designed to eliminate the influence of IL-6, but other clinical indexes, such as ox-LDL, ac-LDL, and LP (a), also have an effect on circulating EPC.

## 5. Conclusion

In conclusion, this study confirmed the relationship between circulating endothelial progenitor cells and the severity of coronary artery disease in unstable angina pectoris, which may be related to the IL-6 level.

## Figures and Tables

**Table 1:** Clinical and biochemical characteristics of the two groups.

Characteristics	Control (*n* = 20)	UA (*n* = 60)
Age (years)	63.3 ± 9.3	66.9 ± 10.2
BMI (kg/cm^2^)	24.7 ± 3.2	24.9 ± 3.3
Heart rate (beats/min)	74.0 ± 12.8	71.6 ± 11.4
Diastolic BP (mmHg)	68.6 ± 5.7	73.9 ± 8.2
Systolic BP (mmHg)	128.7 ± 20.9	132.8 ± 18.4
ALT (mmol/L)	29.6 ± 11.9	30.9 ± 15.5
AST (mmol/L)	31.0 ± 20.7	27.8 ± 14.4
GLU (mmol/L)	8.5 ± 4.5	7.5 ± 3.5
FMD (%)	8.7 ± 1.0	7.0 ± 1.2^*∗*^
IL-6 (pg/ml)	6.7 ± 2.5	11.1 ± 3.1^*∗*^

AST, aspartate aminotransferase; ALT, alanine transaminase; BMI, body mass index; BP, blood pressure; BUN, blood urea nitrogen; Cr, serum creatinine; CRP, C-reactive protein; GLU, glucose; FMD, flow-mediated dilation; IL-6, interleukin-6. Note. Data are given as the mean ± SD. ^*∗*^*P* < 0.05 vs. control.

## Data Availability

The data used to support the findings of this study are available from the corresponding author upon request.
